# Contribution of Different Mechanisms to Ciprofloxacin Resistance in *Salmonella* spp.

**DOI:** 10.3389/fmicb.2021.663731

**Published:** 2021-05-06

**Authors:** Man-Xia Chang, Jin-Fei Zhang, Yin-Huan Sun, Rong-Sheng Li, Xiao-Ling Lin, Ling Yang, Mark A. Webber, Hong-Xia Jiang

**Affiliations:** ^1^Guangdong Key Laboratory of Veterinary Drug Development and Safety Evaluation, College of Veterinary Medicine, South China Agricultural University, Guangzhou, China; ^2^Guangdong Laboratory for Lingnan Modern Agriculture, Guangzhou, China; ^3^Quadram Institute Bioscience, Norwich Research Park, Norwich, United Kingdom; ^4^Norwich Medical School, University of East Anglia, Norwich Research Park, Norwich, United Kingdom

**Keywords:** fluoroquinolone resistance, AcrAB efflux pump, QRDR, PMQR, circular intermediate, *Salmonella*

## Abstract

Development of fluoroquinolone resistance can involve several mechanisms that include chromosomal mutations in genes (*gyrAB* and *parCE*) encoding the target bacterial topoisomerase enzymes, increased expression of the AcrAB-TolC efflux system, and acquisition of transmissible quinolone-resistance genes. In this study, 176 *Salmonella* isolates from animals with a broad range of ciprofloxacin MICs were collected to analyze the contribution of these different mechanisms to different phenotypes. All isolates were classified according to their ciprofloxacin susceptibility pattern into five groups as follows: highly resistant (HR), resistant (R), intermediate (I), reduced susceptibility (RS), and susceptible (S). We found that the ParC T57S substitution was common in strains exhibiting lowest MICs of ciprofloxacin while increased MICs depended on the type of GyrA mutation. The ParC T57S substitution appeared to incur little cost to bacterial fitness on its own. The presence of PMQR genes represented an route for resistance development in the absence of target-site mutations. Switching of the plasmid-mediated quinolone resistance (PMQR) gene location from a plasmid to the chromosome was observed and resulted in decreased ciprofloxacin susceptibility; this also correlated with increased fitness and a stable resistance phenotype. The overexpression of AcrAB-TolC played an important role in isolates with small decreases in susceptibility and expression was upregulated by MarA more often than by RamA. This study increases our understanding of the relative importance of several resistance mechanisms in the development of fluoroquinolone resistance in *Salmonella* from the food chain.

## Introduction

*Salmonella* species are important foodborne pathogens and associated infections can be life-threatening, particularly in elderly and immunocompromised patients (Majowicz et al., 2010). Fluoroquinolones (FQ) are the primary treatment option for life-threatening *Salmonella* infections in clinical practice (Hohmann, 2001). However, the emergence of FQ resistance in *Salmonella* spp. has limited therapeutic options and the World Health Organization categorized FQ-resistant *Salmonella* as a high priority pathogen for the research and development of new antibiotics in 2017 (WHO, 2017).

Mechanisms of FQ resistance include chromosomal mutations in the target enzymes, DNA gyrase and topoisomerase IV, down-regulation of outer membrane porin expression coupled with increases in active drug efflux, as well as acquisition of transmissible quinolone-resistance genes. The development of FQ resistance is often mediated by the accumulation of multiple mutations in a stepwise process and the interplay between multiple resistance mechanisms can result in the development of highly resistant mutants (Redgrave et al., 2014; Hooper and Jacoby, 2015). It has been suggested that, mutation of chromosomally located primary target genes represents the first step in the development of quinolone resistance. For Gram-negative organisms such as *Escherichia coli*, the primary target is typically the GyrA subunit of gyrase. The most common mutation site in the quinolone resistance determining region (QRDR) is serine 83 of GyrA. Highly resistant organisms typically carry a combination of mutations within *gyrA* and *parC* (Machuca et al., 2014; Redgrave et al., 2014; Hooper and Jacoby, 2015). However, whilst similar mutations are often recovered in *Salmonella spp.* as in *E. coli* after quinolone exposure (Marcusson et al., 2009; Machuca et al., 2014), the phenotypic impact resulting from target site mutations seem to have a smaller impact (conferring smaller MIC increases) than in *E. coli* (Heisig, 1996; Lin et al., 2015).

The chromosomal multidrug efflux pump AcrAB-TolC is capable of actively removing FQs and other drugs from the bacterial cell and is known to play an important role in the development of high level FQ resistance (Redgrave et al., 2014),overexpression of *acrAB* has been suggested to be a first step that facilitates high-level resistance development following acquisition of target site mutations (Giraud et al., 2000; Singh et al., 2012).

Transmissible quinolone-resistance mechanisms are often plasmid associated and so are known as “plasmid-mediated quinolone resistance” (PMQR) determinants. These typically confer a low-level of decreased susceptibility to quinolones. As with efflux over-expression, carriage of PMQRs can also facilitate the selection of mutants with higher levels of quinolone resistance through additional chromosomally encoded mechanisms (Hooper and Jacoby, 2015).

There is evidence for clinical quinolone resistance to emerge without target site mutation, a fully resistant *E. coli* strain with a ciprofloxacin MIC of 4 mg/L but without topoisomerase mutations has been reported and was shown to harbor plasmid-mediated *qnrS1* and *oqxAB*, as well as overexpressing *acrAB* and genes encoding for other efflux pumps (Sato et al., 2013). Similarly, an experimentally derived mutant carrying five plasmid copies of *qnrA1* elevated the ciprofloxacin MIC from 0.25 to 2 mg/L; this MIC value exceeds the CLSI breakpoint for resistance (Vinue et al., 2019). In a separate study, several PMQR genes either alone or in combination were reported to mediate ciprofloxacin resistance development in *Salmonella* isolates that did not contain target gene mutations (Lin et al., 2015).

The acquisition of antibiotic resistance in bacteria via either chromosomal or plasmid mechanisms is often accompanied by a fitness cost. But this is not always the case in quinolone resistance development. A study (Marcusson et al., 2009) investigating the fitness effect of various QRDR mutations and efflux activity in *E. coli* revealed that GyrA 83 mutations conferred a fitness gain on the isolates while GyrA 87 substitutions were associated with fitness costs. Acquisition of a triplet of GyrA 83, GyrA 87, and ParC 80 substitutions resulted in fitness gain when efflux was not involved. De-repressed efflux activity was, however, associated with fitness costs. Resistant mutants harboring the same triplet mutations, which was obtained by exposure of a quinolone-susceptible *Salmonella* Typhimurium clinical isolate to increasing concentrations of ciprofloxacin also showed a diminished ability to grow (Fabrega et al., 2014). Huseby et al. (2017) demonstrated that the even low-levels of ciprofloxacin strongly favor growth of topoisomerase mutants and even genotypes (*gyrA* 83 and *parC* 80) which carry a cost in drug free media are favored in sub-inhibitory concentrations of quinolone.

Most of our understanding of the contributions and fitness costs of different mechanisms to quinolone resistance is from laboratory studies or analysis of small numbers of mutants. Given the importance of quinolone resistance as a phenotype, a thorough understanding of the impact of the different FQ resistance mechanisms in the real world is of crucial importance. In the present study, 176 *Salmonella* isolates with a broad range of ciprofloxacin MIC distributions were collected. These isolates were analyzed for correlations between ciprofloxacin resistance phenotypes and: target site mutations, expression levels of *acrAB*, and prevalence of PMQR genes. This research assessed the relative contributions of defined mutant combinations toward fitness in different FQ concentrations and informs how FQ resistance evolves in the food chain.

## Materials and Methods

### Bacterial Strains and Susceptibility Testing

*Salmonella* isolates (126) used in this study were isolated from pork samples in a large-scale slaughterhouse between 2013 and 2014 (Yang et al., 2017). The ciprofloxacin MICs ranged from <0.015 to 64 mg/L although none of these isolates were inhibited by ciprofloxacin concentrations between 0.125 and 2 mg/L. In order to generate a consecutive ciprofloxacin MIC distribution range for each MIC value, we selected 50 additional strains from food-producing animals and raw meat (chicken and pork) as described elsewhere (Zhang et al., 2016; Zhang C.Z. et al., 2019; Yang et al., 2017). This included 11 isolates from swine, 9 from chickens, 17 from ducks and 13 from meat samples (10 chicken and 3 pork) (Table 1). Although efforts were made to screen for strains with a ciprofloxacin MIC between 0.25 and 2 mg/L, these strains were rare. We only identified 3, 3, and 4 isolates in the entire strain collection that possessed ciprofloxacin MICs of 0.5, 1, and 2 mg/L, respectively.

**TABLE 1:** Sources and ciprofloxacin MIC values for *Salmonella* isolates used in this study.

**Sample origin**	**Sampling year**	**No. of isolates possessing the indicated ciprofloxacin MIC (mg/L)**													**Total**
			
		**≤0.015**	**0.03**	**0.06**	**0.125**	**0.25**	**0.5**	**1**	**2**	**4**	**8**	**16**	**32**	**64**	
Pork	2013–2014	28	5	47	0	0	0	0	0	4	29	0	9	4	126
Pork	2016	1	0	0	1	1	0	0	0	0	0	0	0	0	3
Chicken meat	2015	4	1	0	1	0	0	0	0	1	0	0	0	0	10
Swine	2014, 2016	1	1	0	2	1	0	3	4	1	0	0	0	0	11
Chicken	2014, 2016	0	0	0	4	3	0	1	0	0	0	0	2	0	9
Duck	2016	3	0	0	2	0	3	0	0	1	7	1	0	0	17
Total		37	7	47	10	5	3	3	4	7	36	1	11	4	176

The majority of the strains isolated from pork samples were serovars Derby, Rissen and Indiana as previously described (Yang et al., 2017). The 50 additional isolates included Typhimurium (15), Indiana (12), Enteritidis (7), Derby (5), and 11 others.

*Salmonella enterica* subsp. *enterica* serovar Typhimurium SL1344 (MIC_*CIP*_ = 0.015 mg/L) was kindly provided by Prof. Laura Piddock (Institute of Microbiology and Infection, University of Birmingham) and used as a standard reference strain in this study. The MICs of ciprofloxacin were determined by the agar dilution method following CLSI guidelines (CLSI, 2019).

### Detection of Target Gene Mutations and the Presence of PMQR Genes

Identification of mutations in the quinolone resistance determining regions (QRDR) of *gyrA*, *gyrB*, *parC*, and *parE* and identification of *qnr*, *aac(6′)-Ib-cr*, *qepA* and *oqxABR* genes were performed using PCR amplification, primers used are listed in Supplementary Table 1. All PCR amplicons were sequenced by the BGI (Shenzhen, China), and the wild-type *Salmonella* Typhimurium LT2 strain was used as a comparison to identify mutations.

### Detection of the *oqxABR* Circular Intermediates

All *oqxABR*-positive *Salmonella* isolates were screened for the presence of the IS26 insertion sequence (IS), and cyclization of the IS26-*oqxABR* region was determined using primers oqx-IF and oqx-IR. Sequence identity of all PCR amplicons was confirmed by DNA sequence analysis.

### Location of PMQR Genes

The location of PMQR genes and the sizes of PMQR gene-positive plasmids were estimated by *S1*-PFGE and/or *I-CeuI*-PFGE followed by Southern blot hybridization with digoxigenin-dUTP labeled probes of *oqxAB*, *qnrS*, and/or *23S rDNA* genes. Primers used are listed in Supplementary Table 1. Briefly, agarose-embedded DNA for each *Salmonella* strain was digested with 1 U S1 nuclease (Takara Biotechnology, Dalian, China) at 37°C for 45 min or *I-CeuI* (NEB, Ipswich, MA, United States) for 3 h. The restriction fragments were separated by electrophoresis in 0.5 × Tris-borate-EDTA buffer at 14°C for 19 h with pulse times of 2.2 to 54.2 s using a CHEF-MAPPER System (Bio-Rad Laboratories, Hercules, CA, United States). Agarose-embedded DNA from strain H9812 that had been *in situ* digested with *Xba*I (Takara Biotechnology, Dalian, China) at 37°C for 3 h was used as a DNA size marker. The gel was stained with ethidium bromide and DNA bands were visualized under UV light. DNA was transferred to a Hybond-N + membrane for Southern hybridization using conditions suggested by the manufacturer (GE Healthcare, Little Chalfont, United Kingdom). Blots were probed with PMQR genes containing digoxigenin labels using a commercial DIG High Prime DNA Labeling and Detection Starter Kit I (Roche Applied Science, Mannheim, Germany).

### Gene Expression Analysis by qRT-PCR

Bacterial strains were grown in minimal media to an OD_600_ nm of 0.6 and total RNA was isolated using the RNAiso Plus Kit (Takara); mRNAs were reverse transcribed using PrimeScript RT with gDNA Eraser (Takara). The cDNA was quantified using an iQ5 multicolor real-time PCR system (Bio-Rad) using gene-specific primers (Supplementary Table 1), and an iQ SYBR Green Supermix (Bio-Rad). The 16S rRNA gene was used as an internal control gene and relative expression levels of each gene were calculated using the ΔΔCt method and the software provided with the instrument. Data are presented as mean ± SD from three independent assays, in which each RNA sample was tested in triplicate.

### Competitive Fitness Measurements

To directly measure fitness impacts of different resistance mutations we used competition assays between pairs of strains selected to carry different combinations of resistance mechanisms, and with different susceptibility levels to ciprofloxacin. The selected pairs competed against each other were; S(−)/RS(−), RS(c +)/I(p +), I(p +)/R(c +), and I(p +)/HR(p +). In addition, to eradicate any possible strain and serovar specific bias, competition assays were also completed between selected serovar Indiana strains with different resistance mechanisms, RS(−)/HR(p +) (strains HB137/K46) and I(p +)/HR(p +) (strains SP80/K46, and SP80/CL108).

Fitness was analyzed as previously described with some modifications (Zhang C.Z. et al., 2017). Growth competition was determined by pyrosequencing the single nucleotide variations in ParC which distinguished each pair of competed strains. Briefly, the competitive paired strains were mix and co-cultured (1:1 ratio) in antibiotic-free LB broth for 18 h and DNA was extracted using a standard phenol-chloroform-heat method. The DNA from the competitive growth assays was amplified by PCR in triplicate using biotinylated primer pairs targeting the region containing the single nucleotide polymorphism to distinguish the two organisms in the assay by mutations in ParC. All PCR amplifications were visualized on 1% agarose gels prior to pyrosequencing. The purified PCR products were pyrosequenced at BGI. Competition coefficients were defined as the ratio of the strain pairs subjected to different incubation conditions and calculated by measuring percentage yield of the single nucleotide mutations in *parC*. A competition coefficient <1 indicated that the less susceptible strain was more abundant after competition.

## Results

### Assembly and Definition of Study Panel of Isolates

**FIGURE 1:**
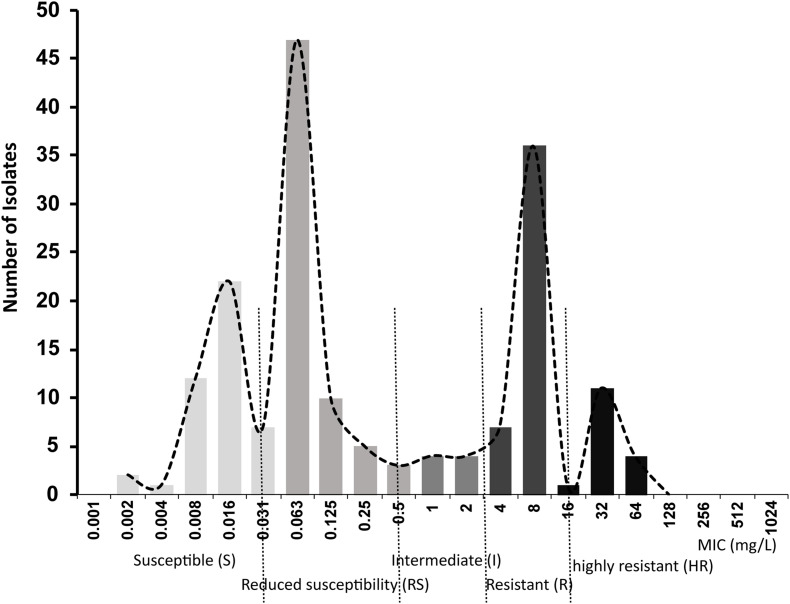
Ciprofloxacin MIC distributions in 176 *Salmonella* strains used in this study.

A primary aim of our study was to identify *Salmonella* isolates with a broad range of ciprofloxacin MICs. We selected 176 *Salmonella* strains from 1,280 isolates, amongst which 91 were susceptible to ciprofloxacin, 20 were deemed intermediate and 65 were resistant according to CLSI breakpoints. However, epidemiologically there were clearly more than three levels of susceptibility in the populations, likely to reflect distinct combinations of resistance mechanisms. Therefore, we applied a 5-level interpretation system using “epidemiological breakpoints.” This system relies on overlapping normal distributions of MIC values (Kronvall, 2010; Zayed et al., 2015). The resulting categories were designated as: highly resistant (HR), resistant (R), intermediate (I), reduced susceptibility (RS), and susceptible (S) (Figure 1). We then randomly selected a total of 32 strains, 5∼7 strains from each group, for detailed study of gene expression and carriage of specific mutations (Table 2 and Supplementary Figure 1).

**TABLE 2:** Characteristics of *Salmonella enterica* isolates from food-producing animals in China.

**Groups**	**Strains**	**Serotype**	**MIC_*CIP*_ (mg/L)**	**QRDR Mutations^ a^**		**mRNA (± SD)^*c*^**						**PMQR genes^*b*^**		
						
				***gyrA***	***parC***	***acrA***	***acrB***	***tolC***	***ramA***	***marA***	***soxS***	***oqxAB***	***aac (6′)-Ib-cr***	**qnrS**
S	M61	Rissen	0.008	–	T57S	10.40 (9.08)	6.61(6.35)	1.32(0.43)	0.47(0.39)	2.5(2.06)	6.71(10.44)	–	–	
	M36	Rissen	0.008	–	T57S	3.96 (3.90)	2.55(2.97)	8.14(8.46)	0.23(0.16)	1.65(1.44)	10.78(17.81)	–	–	
	JH51	NT	0.008	–	T57S	8.72 (9.76)	7.64(9.11)	25.44(23.33)	0.63(0.44)	2.62(3.2)	25.28(42.22)	–	–	
	JH196	NT	0.008	–	T57S	6.72 (5.90)	5.29(7.57)	8.55(9.17)	0.72(0.83)	0.97(0.89)	6.86(10.89)	–	–	
	R17	Rissen	0.008	–	T57S	4.58 (3.13)	5.83(3.73)	0.54(0.36)	0.36(0.33)	4.23(5.46)	1.88(2.03)	–	–	
	A35	Rissen	0.015	–	T57S	10.26 (9.82)	11.84(10.36)	1.83(0.66)	0.78(0.7)	6.55(8.7)	8.47(11.12)	–	–	
	D71	Derby	0.015	–	T57S	40.23 (68.59)	29.72(49.98)	4.69(6.19)	0.88(0.57)	128.1(215.92)	34.74(58.72)	–	–	
RS	M39	Derby	0.06	–	T57S	7.22 (11.53)	6.99(10.2)	25.26(27.39)	0.52(0.43)	44.88(67.58)	14.31(23.17)	C +	–	
	A42	Rissen	0.06	–	T57S	14.41 (23.84)	12.77(19.28)	61.16(80.08)	0.61(0.49)	74.2(110.15)	27.34(44.57)	C +	–	
	J24	Derby	0.06	–	T57S	16.96 (28.80)	14.33(23.41)	63.41(85.18)	0.64(0.57)	81.66(125.27)	28.23(45.73)	C +	–	
	HB137	Indiana	0.25	D87Y	–	14.70 (24.60)	18.79(30.34)	77.49(104.2)	1.66(1.43)	75.06(106.06)	31.07(50.41)	–	–	
	3	Typhimurium	0.5	S83Y	–	15.63 (26.20)	17.39(28.43)	61.87(82.57)	0.88(0.71)	76.44(125.51)	17.07(27.52)	–	–	
	4	Typhimurium	0.5	S83Y	–	24.12 (41.04)	29.5(50.16)	116.38(159.45)	0.58(0.58)	265.95(441.87)	31.27(52.95)	–	–	
I	SP79	Typhimurium	1	D87N	–	1.09 (1.55)	0.59(0.74)	0.33(0.44)	0.38(0.56)	0.57(0.78)	0.24(0.24)	∼180kb P +	∼180kb P +	
	SP128	Typhimurium	1	D87N	–	6.12 (8.28)	6.27(9.02)	0.57(0.76)	2.3(3.37)	89.42(78.44)	2.37(1.82)	–	–	
	SP80	Indiana	1	D87N	–	5.42 (8.42)	6.68(10.81)	6.41(9.94)	0.39(0.27)	20.03(25.57)	1.74(2.32)	∼180kb P +	∼180kb P +	
	SP116	Typhimurium	2	D87N	–	1.06 (0.79)	5.69(8.64)	5.6(8.27)	0.32(0.22)	6.71(6.85)	1.33(1.71)	∼180kb P +	∼180kb P +	
	SP124	Typhimurium	2	D87N	–	0.93 (1.14)	0.7(0.82)	1.28(1.53)	0.36(0.52)	0.61(0.65)	0.23(0.19)	∼180kb P +	∼180kb P +	
R	E39	Derby	4	–	T57S	0.73 (1.03)	0.51(0.59)	1.67(2.14)	0.43(0.65)	0.64(0.62)	0.35(0.29)	C +	C +	C +
	E16	Derby	4	–	T57S	1.03 (1.68)	0.34(0.41)	0.59(0.56)	0.44(0.69)	0.31(0.42)	0.15(0.18)	C +	C +	C +
	F64	Derby	4	–	T57S	0.45 (0.62)	0.32(0.37)	0.58(0.55)	0.54(0.64)	0.77(0.88)	0.24(0.18)	C +	C +	C +
	E9	Derby	8	–	T57S	0.46 (0.50)	0.3(0.27)	0.54(0.53)	0.47(0.59)	0.5(0.62)	0.19(0.1)	C +	C +	C +
	E62	Indiana	8	–	T57S	0.68 (1.07)	0.38(0.46)	0.46(0.42)	0.36(0.35)	0.66(0.62)	0.21(0.1)	C +	C +	C +
	F92	Derby	8	–	T57S	1.05 (1.75)	0.72(1.17)	1.06(0.7)	0.88(0.75)	0.52(0.33)	0.15(0.04)	C +	C +	C +
HR	CL108	Indiana	32	S83F, D87G	T57S, S80R	0.86 (1.43)	0.45(0.7)	0.04(0.03)	5.71(4.87)	2.11(1.32)	0.34(0.37)	C +	C +	C +
	K46	Indiana	32	S83F, D87G	T57S, S80R	3.21 (5.51)	1.14(1.88)	0.12(0.1)	2.46(1.95)	3.83(5.11)	0.23(0.1)	–	∼240kb P +	
	E58	Indiana	64	S83F, D87G	T57S, S80R	6.18 (10.57)	3.61(6.08)	0.27(0.21)	24.95(26.7)	7.42(11.24)	1.69(2.27)	–	∼240kb P +	
	J20	Indiana	64	S83F, D87G	T57S, S80R	2.98 (5.00)	1.04(1.45)	0.11(0.07)	7.62(11.22)	2.33(1.85)	0.31(0.36)	C +	C +	C +
	J46	Indiana	64	S83F, D87G	T57S, S80R	2.03 (3.25)	1.16(1.45)	0.11(0.07)	10.98(16.91)	3.56(3.23)	1.32(1.24)	C +	C +	C +
	E25	Indiana	64	S83F, D87G	T57S, S80R	4.88 (7.84)	3.31(3.86)	0.39(0.27)	42.42(46.68)	9.96(9.58)	3.02(2.99)	–	∼240kb P +	
	E54	Indiana	64	S83F, D87G	T57S, S80R	60.86 (101.91)	50.91(86.83)	5.75(9.87)	59.04(67.8)	234.43(390.32)	46.22(79.61)	–	∼240kb P +	
	B44	Indiana	64	S83F, D87G	T57S, S80R	15.66 (23.54)	8.43(11.93)	0.61(0.77)	18.01(17.75)	44.57(56.69)	1.09(0.99)	–	∼240kb P +	

*−, Wild-type allele (no mutation). ^*a*^QRDR, amino-acids substitutions in Quinolone Resistance Determining Region of topoisomerases encoding genes; *gyrB* and *parE* in all detected strains are all wild-type allele (no mutation). ^*b*^PMQR, Plasmid Mediated Quinolone Resistance; *qnrA, B, C, D* and *qepA* genes cannot be detected in all *Salmonella* isolates; C + : chromosomally located. ^*c*^mRNA levels relative to control genes 16S rRNA, with standard deviations (SD).*

### Correlation of QRDR Target Gene Mutations With MIC Category

Among the isolates that were analyzed, none contained QRDR mutations in *gyrB* and *parE*. In contrast, *parC* mutations that resulted in the ParC T57S substitution were present in S and RS strains with an MIC_*CIP*_ of 0.06 mg/L. ParC T57S was not seen in RS strains when the MIC_*CIP*_ increased to 0.25 and 0.5 mg/L. Isolates with these MICs carried single GyrA S83Y or D87Y substitutions. The “I” strains tested only possessed GyrA D87N single substitutions; interestingly, the ParC T57S substitution was again seen in some R strains, but these lacked GyrA mutations (although had other mechanisms as outlined below). The HR strains all possessed multiple substitutions in both GyrA (S83F/D87G) and ParC (T57S/S80R) (Table 2). The identification of these mutations was positively correlated with their assignment into the 5-level interpretation system.

### The Presence and Location of PMQR Genes

Reduced susceptibility strains (ParC T57S) that possessed MIC_*CIP*_ values of 0.06 mg/L also harbored chromosomal copies of *oqxABR*. PMQR genes were not present in the RS strains that contained the single GyrA substitutions S83Y or D87Y. When the MIC_*CIP*_ levels reached 1–2 mg/L (I strains), the presence of plasmid-borne copies of both *oqxAB* and *aac(6′)-Ib-cr* were detected on ∼180-kb plasmids. In the R strains, *oqxAB*, *aac(6′)-Ib-cr* and *qnr*S were detected and all were located on the chromosome. In HR strains, *aac(6′)-Ib-cr* was found on either ∼240-kb plasmids or was present with *oqxAB* and *qnrS2* on the chromosome (Table 2).

### Expression of AcrAB-TolC and Regulators

We next analyzed whether increases in efflux pump expression correlated with the MIC categories of our isolates. The expression of efflux complex (*acrA, acrB*, and *tolC)* and regulatory (*ramA*, *marA*, and *soxS*) genes for the subset of 32 *Salmonella* isolates were measured (Table 2 and Supplementary Figure 2). Compared with *S.* Typhimurium SL1344, the RS strains exhibited higher expression of *acrAB-tolC* and the regulator *marA* but not *ramA*. Furthermore, the majority of I strains exhibited increased expression of efflux pump genes with concurrent overexpression of *marA*. Surprisingly, the efflux pump genes were not expressed more in R strains compared with *S.* Typhimurium SL1344, whereas there was increased expression of *acrA, acrB, ramA*, and *marA* in HR strains compared with *S.* Typhimurium SL1344. A two-way ANOVA test of expression values for the regulator genes indicated the differences between groups were not likely to be observed by chance (*p* 0.007).

### *oqxABR* Circular Intermediates

**FIGURE 2:**
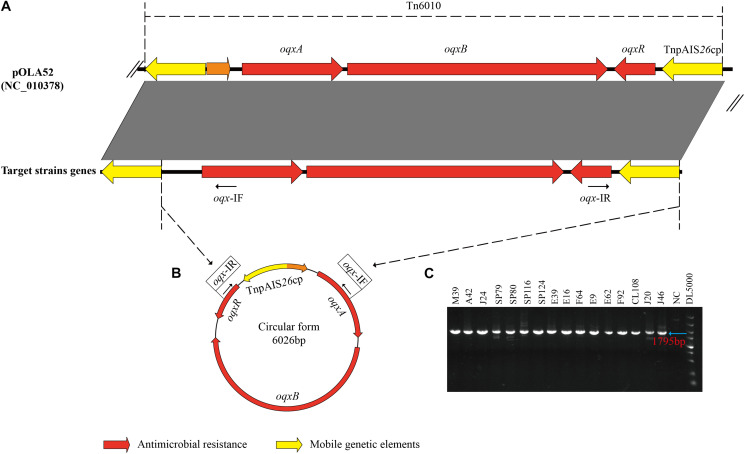
Formation of a circular intermediate by *oqxABR* operon. **(A)** Comparison between genetic structure of transposon Tn6010 located in pOLA52 (accession no. NC_010378) and the *oqxABR* operon positive strains. **(B)** The circular form of Tn6010 and the approximate locations of the reverse primers *oqx-IF* and *oqx-IR* (line arrows). **(C)** Gel electrophoresis of PCR amplicons corresponding to the circular intermediate of Tn6010, detectable in *oqxABR*-positive strains using the reverse primers *oqx-IF* and *oqx-IR*. Primers are listed in Supplementary Table 1. NC, Negative Control.

All 16 *oqxABR*-positive strains possessed IS*26* sequences flanking the *oqxABR* operon (Figure 2A). In addition, all these strains, regardless of whether the genes were located on a plasmid or chromosome, were positive for the presence of circular intermediates containing IS26 and the *oqxABR* genes (Figures 2B,C). Sequencing of this circular intermediate confirmed that the length of the intermediate was 6,026 bp and that *oqxABR* harbored a complete copy of IS26. This intermediate structure was like the *oqxABR* operon present in the widespread composite transposon Tn6010 found in plasmid pOLA52 from *E. coli* (Norman et al., 2008). These results indicate that the Tn6010 element was unstable and prone to excision in our *Salmonella* isolates.

### Competitive Fitness

We compared the competitive fitness of closely related pairs of isolates selected to represent the different combinations of resistance mechanisms and susceptibility levels observed in the whole panel. This included the presence of target gene mutations and presence of PMQR genes on the chromosome (c +) or on plasmids (p +). The competitive growth experiments included the pairs S(−)/RS(−), RS(c +)/I(p +), I(p +)/R(c +), and I(p +)/HR(p +) and Indiana strains RS(−)/HR(p +) (strain HB137/K46) and I(p +)/HR(p +) (strain SP80/K46, and SP80/CL108). All strains used in competition assays are shown in Table 3.

**TABLE 3:** Characteristics of strains used in growth competition assays.

**Strain**	**Susceptibility group^*a*^**	**MIC_*CIP*_ (mg/L)**	**Target mutations**	**PMQR genes**	**Gene location**
M36	S (−)	0.008	ParC T57S	–	ND
M39	RS (c +)	0.06	ParC T57S	*oqxAB*,	chromosome
HB137	RS (−)	0.25	GyrA D87Y	–	ND
SP79	I (p +)	1	GyrA D87N	*oqxAB, aac(6′)-Ib-cr*	plasmid
SP80	I (p +)	1	GyrA D87N	*oqxAB, aac(6′)-Ib-cr*	plasmid
F64	R (c +)	4	ParC T57S	*oqxAB, aac(6′)-Ib-cr and qnrS*	chromosome
K46	HR (p +)	32	GyrA, S83F/D87G ParC, T57S/S80R	*aac(6′)-Ib-cr*	plasmid
CL108	HR(c +)	32	GyrA, S83F/D87G ParC, T57S/S80R	*oqxAB, aac(6′)-Ib-cr and qnrS*	chromosome

*^*a*^−, lacking PMQR; +, PMQR present; p, plasmid location; c, chromosomal location; ND, not detected.*

**FIGURE 3:**
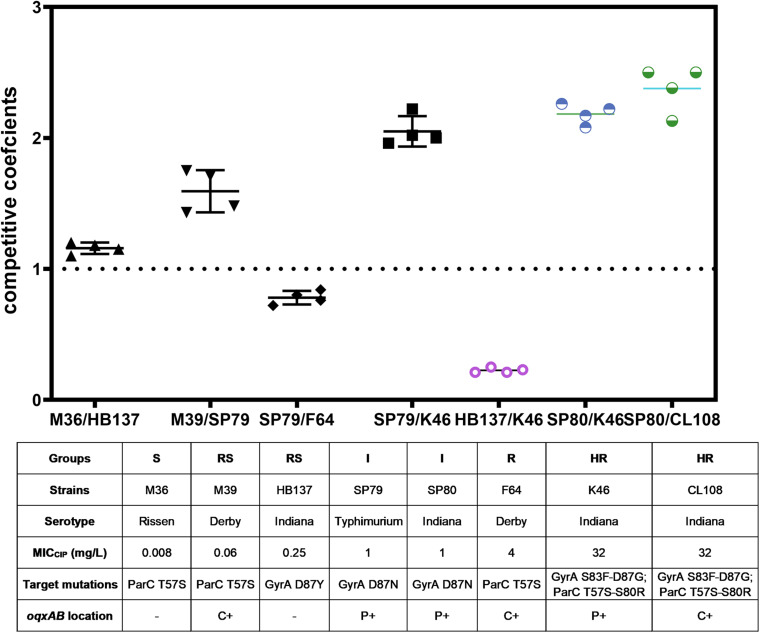
*In vitro* bacterial competition assays. Competition coefficient values were obtained from each independent experiment as indicated. Broken horizontal line, competition coefficient of 1; mean values, short continuous horizontal lines. –, not detected; C+, chromosomally located; P+, plasmid located.

The competition coefficient for the S (−)/RS (−) pair was 1.13, suggesting that the presence of a substitution of GyrA D87Y presented a burden to strain HB137 (RS), while ParC T57S did not impair strain M36 (S). The competition coefficient for RS(c +)/I(p +) counterparts was 1.53, indicating that carriage of both GyrA D87N and the two plasmid PMQR genes produced a burden on strain SP79 (I). The I(p +)/R(c +) competition yielded a competition coefficient of 0.76, indicating the three chromosomal PMQR genes in strain F64 (R) produced less of a burden than when these genes were plasmid-borne. The HR strain K46 carried 2 mutations in GyrA and 2 in ParC. The presence of the plasmid-borne PMQR gene *aac(6′)-Ib-cr* resulted in a competitive disadvantage when compared with the I strain SP79 that possessed a GyrA single mutation; this resulted in a competition coefficient of 1.95 for I(p +)/HR(p +) (Figure 3). The same patterns were seen from the corresponding experiments using identical Indianan strains [I(p +)/HR(p +)], SP80/K46, and SP80/CL108. However, RS strain HB137, harboring a substitution of GyrA D87Y and overexpressing AcrAB, presented a competitive disadvantage when compared with HR strain K46.

## Discussion

The evolution of antimicrobial resistance in bacteria is driven by the pressures exerted following exposure to antimicrobials. However, the impacts of different resistance mechanisms are still not well understood. We analyzed isolates of different *Salmonella* serovars with a broad range of ciprofloxacin MIC distributions in this study. From studying this curated panel of isolates, the current study resulted in the following conclusions:

### ParC T57S Was Present in Isolates With Low Ciprofloxacin MICs

The development of FQ resistance is understood to often require accumulation of multiple mutations in a stepwise process, with mutations that alter GyrA at codon 83 often being considered the “first step” to selection of high-level resistance. Here, we observed a high prevalence of the ParC T57S substitution in strains with lowest MICs of ciprofloxacin (S and RS strains). Whether the ParC T57S substitution contributes to quinolone resistance, is still controversial (Baucheron et al., 2002; Ling et al., 2003; Eaves et al., 2004; Gunell et al., 2009; Zhang W.H. et al., 2017). We found that the presence of ParC T57S was altered as the ciprofloxacin MIC increased; this mutation only appeared alone in strains with MIC_*CIP*_ ≤ 0.06 mg/L. Therefore, we suggest that any impact of this mutation alone of quinolone susceptibility must be low and can only provide a low degree of protection against ciprofloxacin. This change alone may carry little fitness cost which would explain its prevalence in the S and RS strains, however, it may incur a different impact on fitness when associated with GyrA substitutions as no strain carried a single substitution within ParC and any GyrA change in any of the groups. Most resistant strains did carry multiple substitutions within both GyrA and ParC and the specific nature of these changes is likely to impact fitness.

In the intermediate isolates where the MIC_*CIP*_ was higher (I; 4–8 mg/L), strains carried a QRDR mutation, such as S83Y or D87Y/N but the ParC T57S mutation was not present (suggesting this is not a precursor required for development of resistance). ParC T57S was seen in R and HR strains, along with multiple substitutions in both GyrA and ParC, this demonstrates the ParC T57S substitution is viable when in combination with multiple other topoisomerase mutations.

Expression of *acrAB-tol*C was elevated in strains carrying the ParC T57S substitution, but only those where the MIC_*CIP*_ was below the resistance breakpoint. This again suggests that a combination of the ParC T57S change with increased active efflux activity may be important in the early stages of resistance development (where MICs are relatively low). The growth competition assays revealed that the ParC T57S mutation was associated with enhanced bacterial fitness again supporting a low-impact but high-fitness role for this substitution.

### PMQR Genes Were Associated With Ciprofloxacin Resistance Development

Plasmid-mediated quinolone resistance determinants cause decreased susceptibility to FQs while also facilitating the selection of higher levels of FQ resistance (Rodriguez-Martinez et al., 2016). In the present study, *oqxAB* was the most prevalent PMQR gene detected. It was not present in S strains but was first observed in RS strains with an MIC_*CIP*_ 0.06 mg/L and the gene was chromosomally located in all the positive RS strains. The occurrence of *oqxAB* in combination with existing mechanisms, such as ParC T57S and elevated expression of *acrAB-tolC*, were linked to significant increases in MIC_*CIP*_, compared to strains without *oqxAB* but with these other changes. The chromosomal location of *oqxAB* did not appear to incur a fitness cost in strains which also increased *acrAB-tolC* expression and may represent a successful combination of these two mechanisms in terms of susceptibility and fitness. Isolates with an MIC_*CIP*_ of 0.25–0.5 mg/L, also carried mutations in *gyrA*, which imposed a fitness cost.

Some isolates where the MIC_*CIP*_ achieved clinical resistance levels (although without additional mutations in the target genes) carried >2 PMQR genes (*oqxAB, aac(6′)-Ib-cr*, and *qnrS*) combined with mutation in *gyrA*. These genes were found in plasmid locations in intermediate strains (MIC_*CIP*_ 1–2 mg/L) while being observed at chromosomal locations in R strains (MIC_*CIP*_ 4–8 mg/L). Interestingly, the R group did not exhibit any known chromosomal mechanisms, such as target gene mutations or overexpression of *acrAB-tolC* for increased resistance, but did carry three plasmid-borne PMQR genes. This phenomenon is consistent with previous observations where the presence of 2–3 PMQR genes was observed in ciprofloxacin resistant *Salmonella* isolates from pork samples that lacked any target gene mutations (Lin et al., 2015). Our results confirmed that the presence of multiple PMQR genes or multiple copies of PMQR genes can confer resistance to ciprofloxacin.

### The Switch of PMQR Gene Location Between Chromosome and Plasmid Mediated by IS26-oqxAB Correlated With Fitness and Stable Development of Resistance

*OqxAB* is an efflux pump from the RND family that mediates resistance to olaquindox, chloramphenicol and nalidixic acid. The presence of this pump elevates MICs for other antimicrobial reagents including ampicillin and gentamicin (Hansen et al., 2007). The pump is encoded by an operon (*oqxABR*) flanked by IS26 sequences, constituting a composite transposon (Tn6010) (Norman et al., 2008).

The *oqxABR* operon can form a circular structure to facilitate mobilization (He et al., 2015). In this study, we observed that *oqxABR* shifted from chromosomal to plasmid locations in isolates with relatively high MIC_*CIP*_. We performed inverse PCR to determine whether an IS26-*oqxAB* circular intermediate was involved in this process. We found that all 16 *oqxAB*-positive strains possessed the circular intermediate regardless of genomic location. This indicated that the circular intermediate promotes mobilization of this PMQR between chromosome and plasmid. Interestingly, we detected *qnrS* in R strains located on the chromosome with *aac(6′)-Ib-cr* and *oqxAB*. This is consistent with other several studies, where *qnrS co-existing* with *aac(6′)-Ib-cr* and *oqxAB* were frequently detected in *Salmonella spp.* of food animal origin in China (Zhang et al., 2016; Wang et al., 2017; Kuang et al., 2018), and *qnrS* were also detected on the chromosome along with other PMQR genes (Lin et al., 2015). Co-existence of *qnrS* in the chromosome with other genes may be a strategy that confers a relatively high degree of tolerance to FQs without a prohibitive fitness cost which may explain the frequent observation of this combination.

The switch from plasmid to chromosomal location might represent a bacterial strategy for maintaining a resistance trait while adapting to antimicrobial stress (Machuca et al., 2014). Our growth competition assays confirmed that strains carrying PMQR genes on the chromosome had a growth advantage and could outcompete strains possessing PMQR genes on a plasmid. Interestingly, in the majority of our HR strains, only *aac(6′)-Ib-cr* was present. The latter gene was located on a plasmid and its presence coincided with multiple target gene mutations and overexpression of *acrAB*. *aac(6′)-Ib-cr* was associated with a fitness cost, in consistent with Machuca et al. (2014) observation in *E. coli*. Growth competition assays demonstrated that transfer of the *aac(6′)-Ib-cr* gene back to a plasmid location resulted in a fitness cost. Whole genome sequencing data identified a broad host range plasmid IncQ1 carrying no resistance genes in this HR strain. Thus, it is likely that the presence of this plasmid played a role in *aac(6′)-Ib-cr* gene movement from chromosome to plasmid, which has been previously suggested (Loftie-Eaton and Rawlings, 2012; Barry et al., 2019; Chen et al., 2019).

### Increased Expression of *acrAB-tolC* Plays a Role in Isolates With Low Ciprofloxacin MICs via *marA* Rather Than *ramA*

Increased drug efflux primarily occurs *via* overexpression of AcrAB-TolC, the primary efflux pump, in Enterobacteriaceae and drug efflux is a primary mechanism for quinolone resistance in *Salmonella* (Baucheron et al., 2004; Blair et al., 2009, 2014). In accordance with studies that analyzed experimentally derived ciprofloxacin mutants (Redgrave et al., 2014; Hooper and Jacoby, 2015), we observed elevated expression of AcrAB in the early stages of resistance development prior to the occurrence of *gyrA* mutations (Vidovic et al., 2019). Significant levels of *acrAB-tolC* were observed in the RS strains.

Multidrug efflux pumps are expressed under precise transcriptional control. In *Salmonella*, RamA is often considered the master regulator of *acrAB* (Ricci et al., 2014). We did not detect the overexpression of *ramA* in strains that expressed high levels of *acrAB* but had relatively low ciprofloxacin MICs (Groups S and RS). Instead, this overexpression was accompanied by increases in the expression of *marA*. A similar observation was made with laboratory selected ciprofloxacin-resistant mutants where mutation of *ramR* was mainly seen in mutants with very high ciprofloxacin MICs (Vidovic et al., 2019). Interestingly, efflux pump genes were not expressed more in R strains compared with *S*. Typhimurium SL1344, in agreement with earlier observations made with MDR *Klebsiella* and MDR *E. coli* that strains capable of evolving multiple energetically favorable QRDR mutations will use less efflux than isolates with fewer favorable QRDR alterations (Toth et al., 2014; Johnson et al., 2015). The overexpression of *ramA* was only detected in highly resistant strains. Our data suggests that whilst both MarA and RamA can up-regulate *acrAB*, there is a preference for utilizing MarA in less resistant strains and RamA in the most resistant strains. Both MarA and RamA are pleiotropic regulators and the overexpression of *ramA* may play additional roles as well as regulating *acrAB* in highly resistant strains more compatible with this phenotype than over-expression of *marA*. This is in line with the previous research in which a deletion in the RamR-binding sites in the *ramA* promoter correlated with highest expression levels of efflux pumps (Fabrega et al., 2016).

**FIGURE 4:**
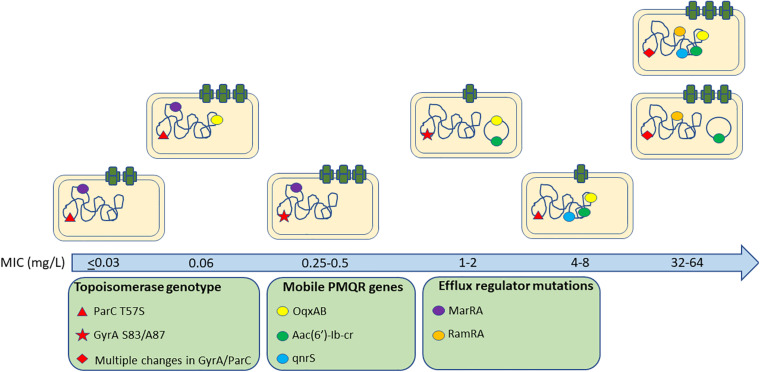
Quinolone resistance genes associated with different ciprofloxacin MICs amongst isolates.

In conclusion, we observed a complex interplay between a pool of quinolone resistance mechanisms, their contribution to susceptibility and fitness costs (Figure 4). In general, we found no significant differences between different serovars of *Salmonella* suggesting common impacts from these genotypes across the genus although only *S.* Indiana isolates were found in the HR group suggesting they may be more able to develop high level resistance. Whilst natural variations in genes related to phylogeny may mask impacts on resistance, the topoisomerase genes and other known quinolone associated resistance pathways are highly conserved across the *Salmonella* genus and studying a large and diverse panel of real-world isolates can be informative for mutations circulating in practice which have been selected in real conditions.

We suggest an important role for substitutions within ParC and *acrAB* expression in the early stages of ciprofloxacin resistance development prior to the occurrence of *gyrA* mutations. Highly resistant isolates acquire multiple target site mutations and PMQR determinants although these are often integrated in the chromosome to minimize fitness costs and expression of *acrAB-tolC* is returned to wild-type levels, probably for the same reason. Whilst we are able here to identify genotypes which appear to be fit in conferring different levels of ciprofloxacin tolerance, it is still not possible to precisely assign a universal pathway in relation to the selection of resistance. Understanding the relative fitness of different genotypes in different selective conditions will help predict the evolutionary trajectory of AMR while also helping us to understand how resistance emerges in complex situations where multiple genes collaborate to confer resistance.

## Data Availability Statement

The raw data supporting the conclusions of this article will be made available by the authors, without undue reservation.

## Author Contributions

H-XJ conceived and designed the experiments. M-XC, J-FZ, Y-HS, X-LL, and R-SL performed the experiments. M-XC, J-FZ, MW, and H-XJ analyzed the data. M-XC, Y-HS, and H-XJ contributed reagents, materials, and analyses tools. M-XC, LY, MW, and H-XJ wrote and revised the manuscript. All authors contributed to the article and approved the submitted version.

## Conflict of Interest

The authors declare that the research was conducted in the absence of any commercial or financial relationships that could be construed as a potential conflict of interest.
