# Supplementary material for: Contribution of Different Mechanisms to Ciprofloxacin Resistance in Salmonella spp
Source: Front Microbiol. 2021 May 6;12:663731. doi: 10.3389/fmicb.2021.663731 (PMC8137344; doi:10.3389/fmicb.2021.663731)
Supplement: Supplementary file 1 [file Data_Sheet_1.PDF]

Supplementary Figures

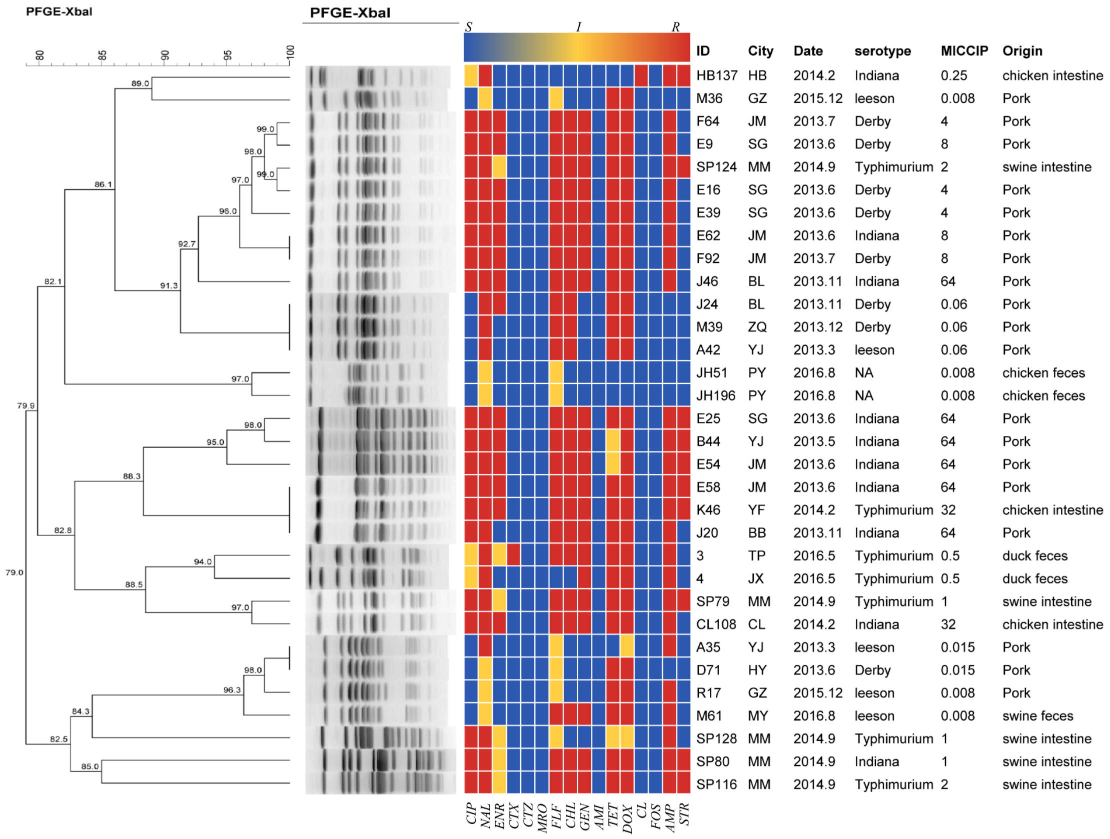

Figure S1. PFGE patterns and antimicrobial resistance profiles of the 32 Salmonella isolates. Dendrograms of XbaI-PFGE are presented on the left, and the results of the antimicrobial susceptibility tests are shown to be aligned with the dendrograms. Red indicates resistance(R) to the corresponding antimicrobials, Orange yellow indicates intermediate susceptibility (I), and blue indicates susceptibility(S).

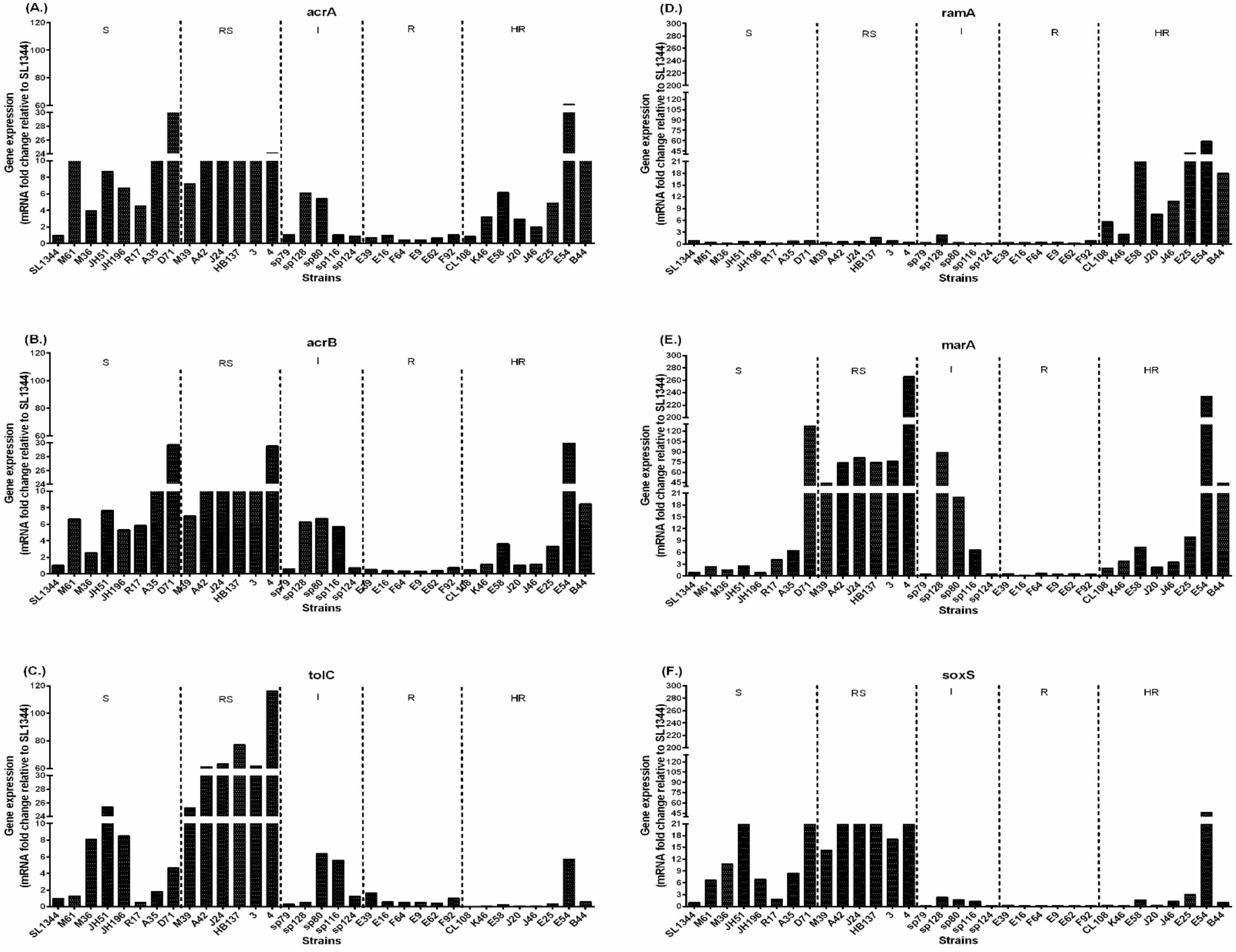
(A.)
acrA
S
RS
I
R
HR
Gene expression (mRNA fold change relative to SL1344)
SL1344 M61 M36 JH51 JH196 R17 A35 D71 M39 A42 J24 HB137 3 4 sp79 sp128 sp80 sp116 sp124 E39 E16 F64 E9 E62 F92 CL108 K46 E58 J20 J46 E25 E54 B44
Strains
(B.)
acrB
S
RS
I
R
HR
Gene expression (mRNA fold change relative to SL1344)
Strains
(C.)
tolC
S
RS
I
R
HR
Gene expression (mRNA fold change relative to SL1344)
Strains
(D.)
ramA
S
RS
I
R
HR
Gene expression (mRNA fold change relative to SL1344)
Strains
(E.)
marA
S
RS
I
R
HR
Gene expression (mRNA fold change relative to SL1344)
Strains
(F.)
soxS
S
RS
I
R
HR
Gene expression (mRNA fold change relative to SL1344)
Strains

Figure S2. Levels of mRNA of (A-C) efflux pump genes and (D-F) regulators in test strains.

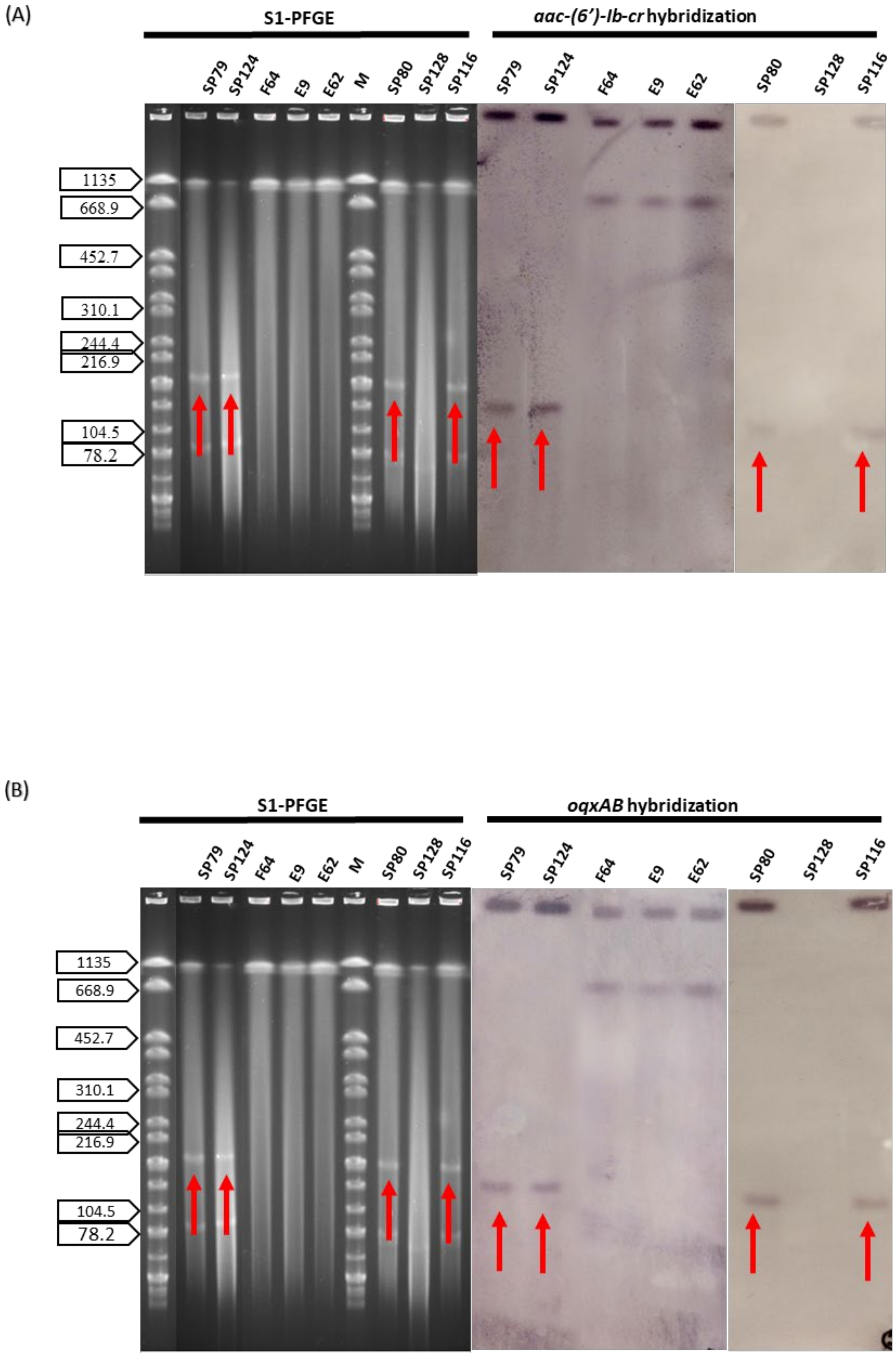
(A)
S1-PFGE
aac-(6')-Ib-cr hybridization
SP79
SP124
F64
E9
E62
M
SP80
SP128
SP116
1135
668.9
452.7
310.1
244.4
216.9
104.5
78.2
(B)
S1-PFGE
oqxAB hybridization
SP79
SP124
F64
E9
E62
M
SP80
SP128
SP116
1135
668.9
452.7
310.1
244.4
216.9
104.5
78.2

(C)

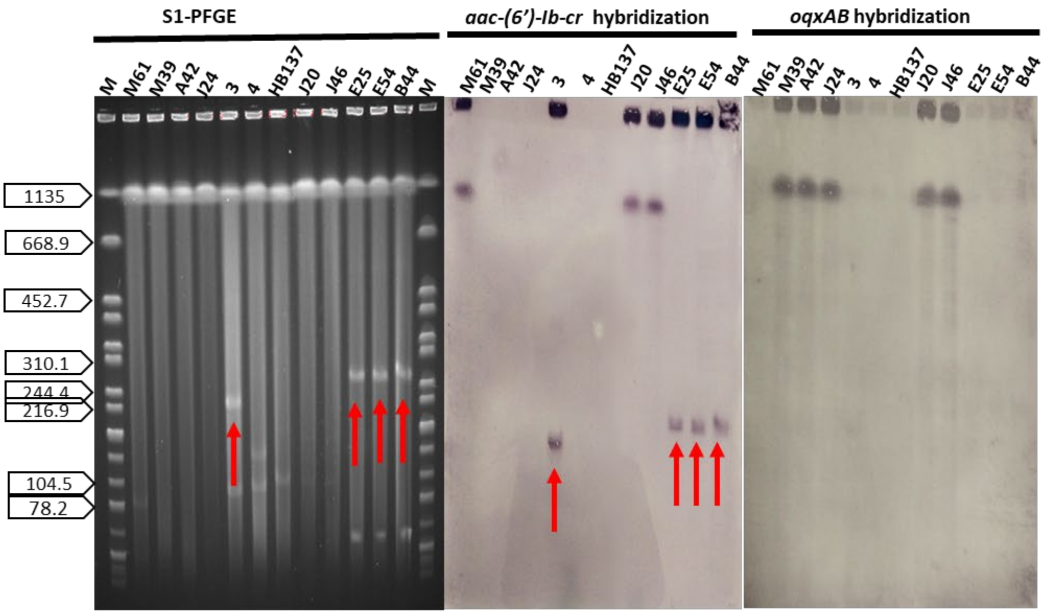

S1-PFGE
aac-(6')-Ib-cr hybridization
oqxAB hybridization
M M61 M39 A42 J24 3 4 HB137 J20 J46 E25 E54 B44 M
M61 M39 A42 J24 3 4 HB137 J20 J46 E25 E54 B44
M61 M39 A42 J24 3 4 HB137 J20 J46 E25 E54 B44
1135
668.9
452.7
310.1
244.4
216.9
104.5
78.2

(D)

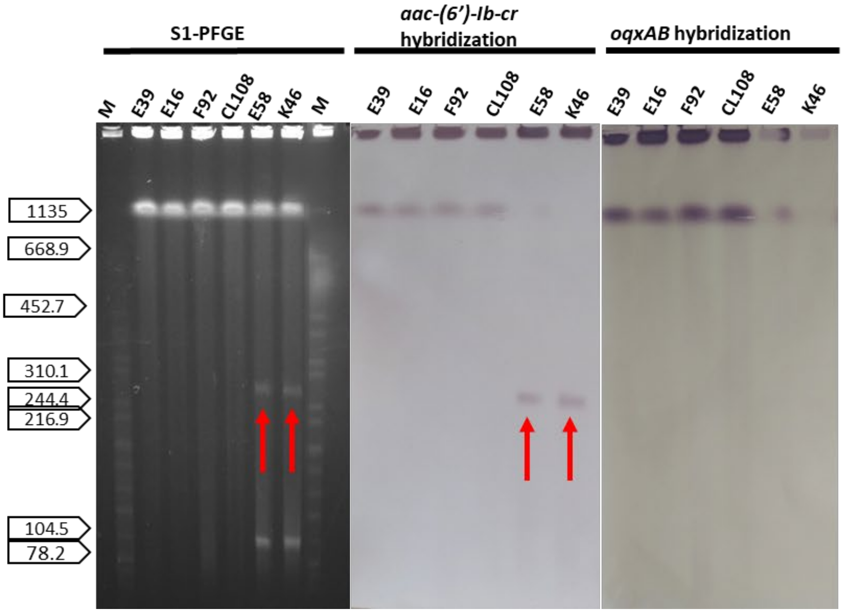

S1-PFGE
aac-(6')-Ib-cr hybridization
oqxAB hybridization
M E39 E16 F92 CL108 E58 K46 M
E39 E16 F92 CL108 E58 K46
E39 E16 F92 CL108 E58 K46
1135
668.9
452.7
310.1
244.4
216.9
104.5
78.2

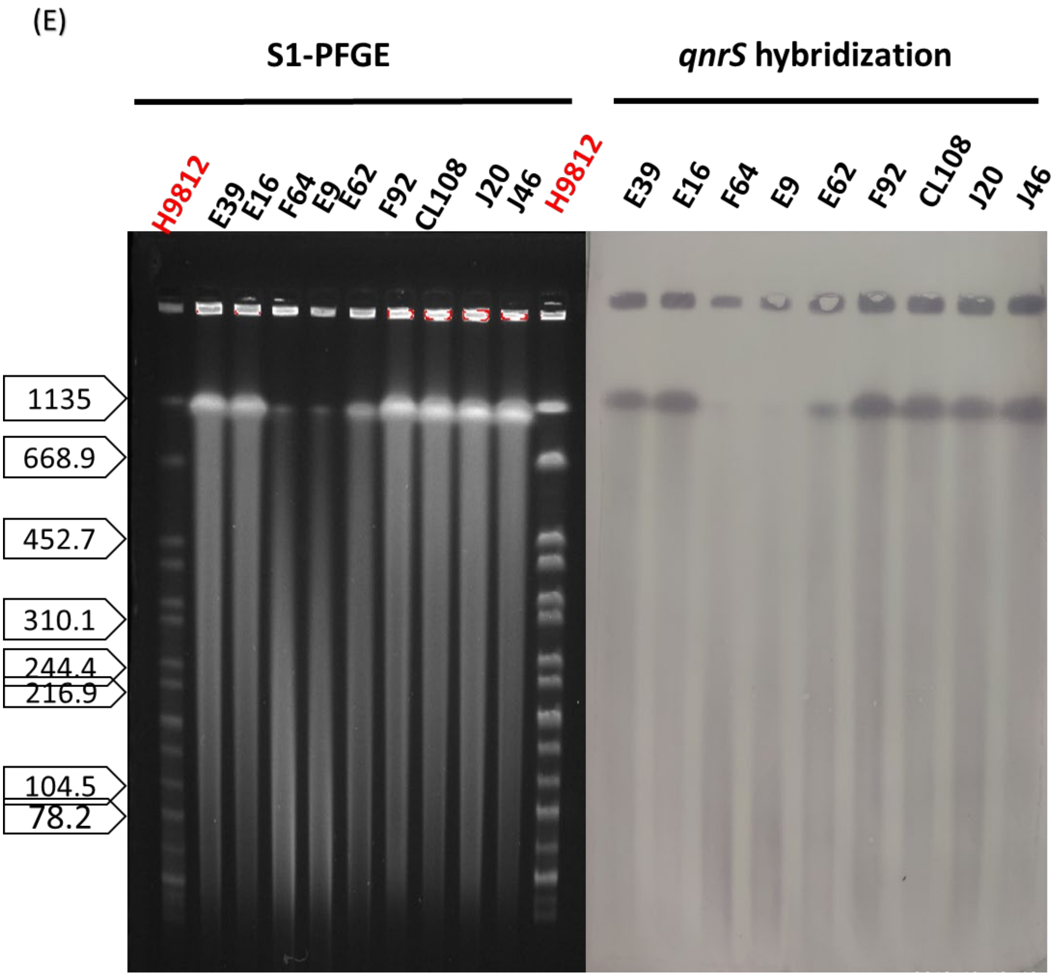

Figure S3. S1-PFGE of S1 nuclease-digested genomic DNA and Southern blot hybridization with the corresponding PMQR probe. The arrow indicates plasmids in the isolates.

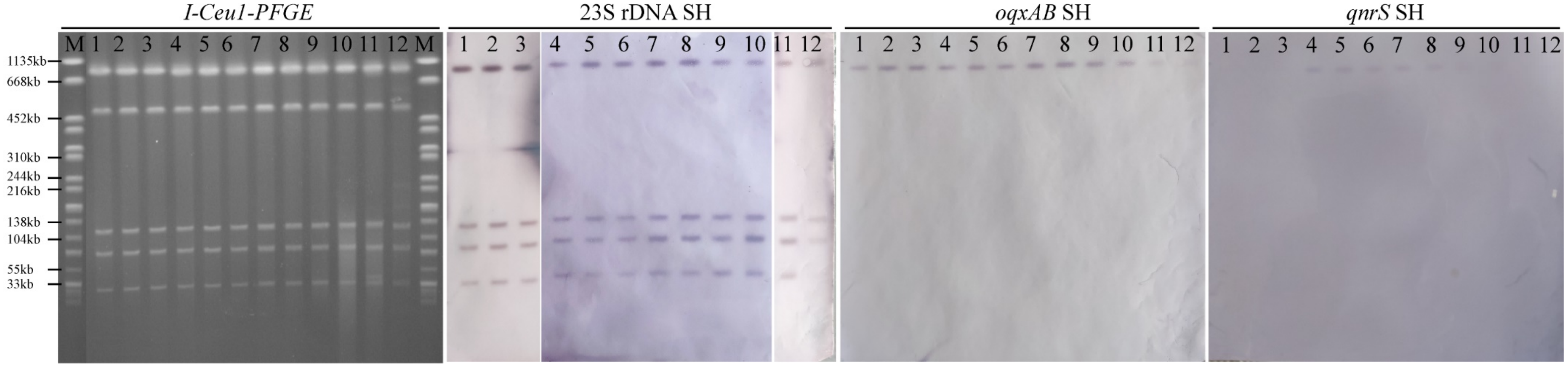

Figure S4. Chromosomal location of *oqxAB* and *qnrS* in the Salmonella isolates using *I-Ceu1*-PFGE and Southern hybridization with 23S rDNA, *oqxAB* and *qnrS* probes. lane 1, M39; lane 2, A42; lane 3, J24; lane 4, E39; lane 5, E16; lane 6, F64; lane 7, E9; lane 8, E62; lane 9, F92; lane 10, CL108; lane 11, J20; lane 12, J46; M, H9812 as a Marker. SH, Southern hybridization using the 23S rDNA, *oqxAB* or *qnrS* probes.

Supplementary Table

Oligonucleotide primers and annealing temperature (Ta) used in this study.

Primers name	Primer sequences（5′-3′）	Ta (°C)	Product sizes（bp）	references
**Target genes**				
gyrA	F：ATGAGCGACCTTGCGAGAGA<br>R：TTACTCGTCAGCGTCATCCGCA	60	2637	This study
gyrB	F：ATGTCGAATTCTTATGACTCCTCC<br>R：TTAAATATCGATATTCGCTGCTTTC	55	2415	
parC	F：TTCCTCGCCACCTGTCTCA<br>R：TTGGTAAGTGTGGCTCTCTG	58	2563	
parE	F：ATGACGCAAACTTATAACGCTGA<br>R：TTACACGTCGAGATCCGCG	56	1893	
**RT-PCR**				
acrA	F：AAAACGGCAAAGCGAAGGT<br>R：GTACCGGACTGCGGGAATT	59	178	(Zhang et al., 2017)
acrB	F：TGAAAAAAATGGAACCGTTCTTC<br>R：CGAACGGCGTGGTGTCA	58	184	
tolC	F：GCCCGTGCGCAATATGAT<br>R：CCGCGTTATCCAGGTTGTTG	58	243	
ramA	F：CACGATTGTCGAGTGGATTG<br>R：AAAATGCGCGTAAAGGTTTG	58	232	
soxS	F：AAATCGGGCTACTCCAAGTG<br>R：CTACAGGCGGTGACGGTAAT	59	217	
marA	F：ATCCGCAGCCGTAAAATGAC<br>R：TGGTTCAGCGGCAGCATATA	59	180	
16S	F：GGTGTAGCGGTGAAATGCGTAG<br>R：CCAGGACACAACCTCCAAGT	58	163	
**PMQR detected and for probes**				
qnrA	F: ATTTCTCA CGCCAGGATTTG<br>R: GATCGGCAAAGGTTAGGTCA	53	516	(Li et al., 2013)
qnrB	F: GATCGTGAAAGCCAGAAAGG<br>R: ACGATGCCTGGTAGTTGTCC	53	469	
qnrC	F: GGGTTGTACATTTATTGAATC<br>R: TCCACTTTACGAGGTTCT	50	447	
qnrD	F: CGAGATCAATTTACGGGGAATA<br>R: AACAAGCTAGAGCGCCTG	50	500~600	
qnrS	F: ACGACATTCGTCAACTGCAA<br>R: TAAATTGGCACCCTGTAGGC	53	417	
qepA	F: CGGCGGCGTGTTGCTGGAGTTCTT<br>R: CCGACAGGCCCACGACGAGGATGC	60	548	
aac(6′)-Ib-cr	F: TTGCGATGCTCTATGAGTGGCTA<br>R: CTCGAATGCCTGGCGTGTTT	55	482	
oqxA	F: AGTCCATACCAACCTCGTCTCC<br>R: GCGTGGCTTTGAACTCTGC	55	529	
oqxB	F: CATTGGCGGCGTGAAGA<br>R: CCTGATTATTGCGGGTGC	55	637	
***23S rDNA* for probe**				

23S rDNA	F: AATGATGGCCAGGCTGTCTCC<br>R: CCGCCGTCGATATGAACTCTTG	60	512	(Liu and Sanderson, 1995)
**Genetic environment**				
IS*26*	Fc：CTCCTCCCGTCGTAACAGC<br>R：AAACTCTGCTTACCAGGCGC	60	377	
IS*26-oqxA*	F：GCTGTTACGACGGGAGGAG<br>R_A：GGAGACGAGGTTGGTATGGA	57	1078	This study
IS*26-oqxR*	Fc：CTCCTCCCGTCGTAACAGC<br>R_R：CCGAAAGATAGATATCACGCAGGGT	60	736	
**Reverse PCR primers for oqxAB circular form**				
oqx	IF：GGAGACGAGGTTGGTATGGA<br>IR：CCGAAAGATAGATATCACGCAGGGT	57	1795	This study

F: forward; R: reverse；Ta: annealing temperature

Li, L., Liao, X., Yang, Y., Sun, J., Li, L., Liu, B., et al. (2013). Spread of oqxAB in Salmonella enterica serotype Typhimurium predominantly by IncHI2 plasmids. *J Antimicrob Chemother* 68(10)**,** 2263-2268. doi: 10.1093/jac/dkt209.

Liu, S.L., and Sanderson, K.E. (1995). I-CeuI reveals conservation of the genome of independent strains of Salmonella typhimurium. *J Bacteriol* 177(11)**,** 3355-3357. doi: 10.1128/jb.177.11.3355-3357.1995.

Zhang, C.Z., Ren, S.Q., Chang, M.X., Chen, P.X., Ding, H.Z., and Jiang, H.X. (2017). Resistance mechanisms and fitness of Salmonella Typhimurium and Salmonella Enteritidis mutants evolved under selection with ciprofloxacin in vitro. *Sci Rep* 7(1)**,** 9113. doi: 10.1038/s41598-017-09151-y.
